# Weaker Connectivity of the Cortical Networks Is Linked with the Uncharacteristic Gait in Youth with Cerebral Palsy

**DOI:** 10.3390/brainsci11081065

**Published:** 2021-08-13

**Authors:** Gaelle E. Doucet, Sarah Baker, Tony W. Wilson, Max J. Kurz

**Affiliations:** Institute for Human Neuroscience, Boys Town National Research Hospital, 14090 Mother Teresa Lane, Boys Town, NE 68010, USA; gaelle.doucet@boystown.org (G.E.D.); sarah.baker@boystown.org (S.B.); tony.wilson@boystown.org (T.W.W.)

**Keywords:** walking, resting-state fMRI, sensorimotor network, visual network

## Abstract

Cerebral palsy (CP) is the most prevalent pediatric neurologic impairment and is associated with major mobility deficiencies. This has led to extensive investigations of the sensorimotor network, with far less research focusing on other major networks. The aim of this study was to investigate the functional connectivity (FC) of the main sensory networks (i.e., visual and auditory) and the sensorimotor network, and to link FC to the gait biomechanics of youth with CP. Using resting-state functional magnetic resonance imaging, we first identified the sensorimotor, visual and auditory networks in youth with CP and neurotypical controls. Our analysis revealed reduced FC among the networks in the youth with CP relative to the controls. Notably, the visual network showed lower FC with both the sensorimotor and auditory networks. Furthermore, higher FC between the visual and sensorimotor cortices was associated with larger step length (r = 0.74, p_FDR_ = 0.04) in youth with CP. These results confirm that CP is associated with functional brain abnormalities beyond the sensorimotor network, suggesting abnormal functional integration of the brain’s motor and primary sensory systems. The significant association between abnormal visuo-motor FC and gait could indicate a link with visuomotor disorders in this patient population.

## 1. Introduction

Cerebral palsy (CP) is the most prevalent pediatric neurologic impairment diagnosed in the United States and often results in lifelong mobility challenges [1]. The breadth of the muscular performance and sensory acuity deficiencies in youth with CP have largely fueled the impression that the altered spatiotemporal gait biomechanics primarily originate from aberrations in sensorimotor cortical activity [2,3,4,5]. Despite this impression, a growing body of literature has highlighted that youth with CP also have altered activity in the primary occipital cortices and visual MT areas while processing visual stimuli [6,7,8]. These studies imply that the perinatal insult likely impacts several key brain networks that expend beyond the sensorimotor cortices. However, the possible connection between the altered sensorimotor activity and other key brain networks has not been widely considered.

Resting-state functional magnetic resonance imaging (rs-fMRI) has been increasingly used to reveal the integrity of brain networks [9,10,11,12]. The spatiotemporal configuration of resting-state networks (RSNs) is based on their functional connectivity (FC), which represents the temporal correlation of the blood oxygen level-dependent signals between their constituent brain regions [9]. This technique has been instrumental in mapping the brain functional connectome and how networks remain interconnected [11,12]. Our previous work and other studies have described that the brain networks can be divided into two opposite systems supporting different mental processes: the intrinsic system, involved in internally guided, higher-order mental functions, and the extrinsic system, supporting externally driven, specialized sensory and motor processing [11,12,13]. This latter is composed of the networks largely covering the primary cortices, namely the sensorimotor (SMN), auditory (AUD) and visual (VIS) networks, which have been shown to be strongly positively correlated in healthy populations [11,13]. Furthermore, each of these brain networks relies on established white matter pathways [14,15,16], has been consistently and robustly defined by its spatiotemporal configuration and functional roles [17,18,19,20], and shows relatively low interindividual variability in anatomical morphology [21] and in resting-state FC [22,23,24].

Over the past decade, rs-fMRI has provided unique insights on how the perinatal brain injuries in youth with CP impact the integrity of the RSNs [25,26,27,28,29]. These studies have revealed that youth with a spastic diplegic presentation have higher FC within the sensorimotor cortices than their neurotypical peers [25,26]. In contrast, FC between the sensorimotor cortex and the cerebellum, contralateral sensorimotor cortex, cingulate motor area, visual cortices and parietal lobes have been described as weaker in youth with CP compared to controls [25,28,30,31]. Furthermore, the strength of the respective connections tends to be reduced even further in those with more severe gross motor presentations [28,29]. Conversely, youth with a hemiplegic presentation that has resulted from hemispheric specific volume loss due to a stroke tend to display stronger FC within the sensorimotor cortices and default-mode network than controls [27,32]. These youth also tend to display more diffuse activity in the sensorimotor area of the hemisphere without volume loss when compared with the hemisphere that has incurred the perinatal vascular insult [29,33]. Altogether, there is mounting evidence that the developmental brain injuries in youth with CP disturb the integrity of the RSNs.

Despite the field’s growing understanding of RSNs in youth with CP, it is currently unclear whether alterations in FC among the SMN and other primary networks (e.g., auditory and visual) are linked to the extent of mobility deficits. The purpose of this investigation was to use rs-fMRI to map the SMN, AUD and VIS networks in youth with CP, and to determine if the FC emerging from these RSNs is linked with their altered spatiotemporal gait biomechanics. Based on prior rs-fMRI work [26,28], we hypothesized that the youth with CP would display weaker FC for the SMN, AUD, and VIS networks. Lastly, we hypothesized that youth with CP who have weaker FC in the SMN and VIS networks would be more likely to have greater alterations in their spatiotemporal gait biomechanics. Conversely, since hearing is not a primary factor for gait, we speculated that the strength of FC in the AUD network would not be related to the altered spatiotemporal gait biomechanics seen in youth with CP.

## 2. Material and Method

### 2.1. Participants

In total, 65 participants were included in the study. Of these participants, 27 were youth with CP that had a spastic presentation (mean (SD) age: 16.39 (4.90) years, Gross Motor Function Classification Scale (GMFCS) levels: I-IV), and 38 demographically matched neurotypical controls (mean (SD) age: 14.44 (2.35) years) (Table 1). Youth with CP were excluded from the study if they had an orthopedic surgery or anti-spasticity treatments within the last 6 months, dorsal rhizotomy, metal in their body that would preclude an MRI and/or clinical diagnosis of an arterial ischemic stroke or middle cerebral artery stroke. The respective stroke cases were excluded, as they are associated with a large hemisphere specific volume loss that has previously been reported to have different effects on the RSN FC than the other structural insults in youth with CP with spastic diplegic presentation [27,32,33]. The Institutional Review Board reviewed and approved the protocol for this investigation. All parents provided written consent that their child could participate in the investigation, and the youth assented.

### 2.2. MRI Data Acquisition

All participants underwent an MRI scan on a 3-T Siemens Skyra MRI scanner using a 32-channel head coil. High-resolution T1-weighted images were collected using an MPRAGE sequence (192 slices, TR = 2400 ms, TE = 1.94 ms, FOV = 256 mm, flip angle = 8°, voxel size = 1 × 1 × 1 mm). The rs-fMRI sequence was collected using a single-shot echoplanar gradient echo imaging sequence acquiring a T2* signal with the following parameters: 645 volumes, 48 axial slices acquired parallel to the AC-PC line, TR = 0.46 s, TE = 29 ms, FOV = 82 mm, flip angle = 44°. The in-plane resolution was 3.3 × 3.3 mm^2^, and the slice thickness was 3 mm. The participants were instructed to keep their eyes open and focus their gaze on a fixation cross while they remained still throughout the scan.

### 2.3. Preprocessing Analyses

The rs-fMRI data were preprocessed using SPM12 and the DPABI Toolbox [34]. Preprocessing procedures included removal of the first 5 volumes, motion correction to the first volume with rigid-body alignment, co-registration between the functional scans and the anatomical T1-weighted scan, linear detrending, and regression of motion parameters and their derivatives (24-parameter model) [35], as well as white matter (WM) and cerebrospinal fluid (CSF) timeseries. The WM and CSF signals were computed using a component-based noise reduction method (CompCor, 5 principal components) [36]. Spatial normalization of the functional images into Montreal Neurological Institute (MNI) stereotaxic standard space, and spatial smoothing with a full width of 6 mm full width at half-maximum Gaussian kernel, were then conducted. Last, bandpass filtering was applied at 0.01–0.1 Hz [37].

### 2.4. Quality Control

Each participant’s structural scan was individually reviewed. None of the participants had visible volume loss that would have affected the integrity of the cortical surface in neural areas of interest (i.e., primary cortices). However, we excluded eight participants with CP and one control who had excessive head movement based on maximum transient (volume-to-volume) head motion above 3 mm translation or degree rotation during the rs-fMRI scan. After exclusion, head motion did not differ between the two groups (mean framewise displacement: *p* = 0.1). To ensure that the results were not related to head motion, mean framewise displacement was added as a covariate of no interest in all group analyses (see below).

### 2.5. Seed-Based Functional Connectivity Analyses

#### 2.5.1. Definition of the Seed Regions

To map the sensorimotor, visual and auditory networks, we selected three previously validated seed regions [38,39] and located these regions in the left precentral cortex (MNI coordinates: x = −41, y = −20, z = 62), left lingual gyrus (MNI coordinates: x = −16, y = −74, z = −7) and left Heschl gyrus (MNI coordinates: x = −40, y = −24, z = 9), respectively. We created a 6 mm-radius sphere centered on each coordinate and calculated the mean signal time course for each seed and each participant. Each participant’s anatomical scan was individually checked to ensure that the seed covered the expected gyrus and was located in a grey matter area.

#### 2.5.2. First-Level Analyses

For each participant, a correlation map was created by computing the correlation between the mean time course from each seed and the time course from all other brain voxels. Next, these maps were submitted to a Fisher r-to-Z transformation (Z(r)). All further analyses were conducted on these transformed data.

#### 2.5.3. Second-Level Analyses

First, we validated the location of each seed by identifying the sensorimotor, visual and auditory networks within the participants. For each seed separately, the individual Z(r) values maps were entered into a one-sample *t*-test, adding age, sex and mean framewise displacement as covariates of no interest. Each independent test resulted in a spatial map for each seed. A height threshold at *p* < 0.0001 (uncorrected, cluster size > 50 voxels) was chosen. Second, individual Z(r) values maps were entered into a second-level random-effects analyses to determine if differences in FC were present between the two diagnostic groups. Significant differences were considered at a whole-brain height threshold fixed at *p* < 0.001 (uncorrected), and the spatial extent consistent with image smoothness and the expected number of voxels per cluster was utilized (k > 20). In each analysis, age, sex and mean framewise displacement were added as covariates of no interest. If the two-sample *t*-test results associated with a seed region showed significant clusters, these clusters were extracted as regions of interest (ROIs). The mean timeseries of these ROIs were then computed.

### 2.6. Spatiotemporal Gait Biomechanics

The spatiotemporal gait biomechanics of the participants while walking at a preferred speed were assessed with a GAITRite digital mat (CIR systems, Franklin, NJ, USA). The participant’s walking velocity, step width, cadence and step length were quantified from two walking trials with an average of 6 steps each. The participants with CP used a walker or forearm crutches if needed. The trace of the assistive device was later removed using the GAITRite mat software. The average of the respective trials was used as the primary outcome variable, and separate independent *t*-tests were used to evaluate the group differences at the 0.05 alpha level.

To determine the potential association between the abnormal RSN FC and gait in the youth with CP, the individual Z(r) computed between the seed region and each ROI identified during the second-level analyses were correlated with the respective spatiotemporal gait biomechanics using nonparametric Spearman’s correlation analyses. Significant results were reported at a significant level of at *p* < 0.05 after applying a False-Discovery Rate (FDR) correction.

## 3. Results

### 3.1. Demographics

After quality control, the final samples were 19 youth with CP (mean age (std) = 16.93 (4.90); GMFCS level I: n = 6; II, n = 7, III: n = 3, IV: n = 3) and 37 neurotypical controls (mean age (std) = 14.51 (2.35)). Among the 19 youth with CP, 14 (74%) had spastic diplegic presentation and 5 (26%) had hemiplegic presentation. No significant difference in age (*p* = 0.054) or sex (*p* = 0.2) was found between the controls and youth with CP.

### 3.2. Sensorimotor Network Functional Connectivity

**Figure 1:**
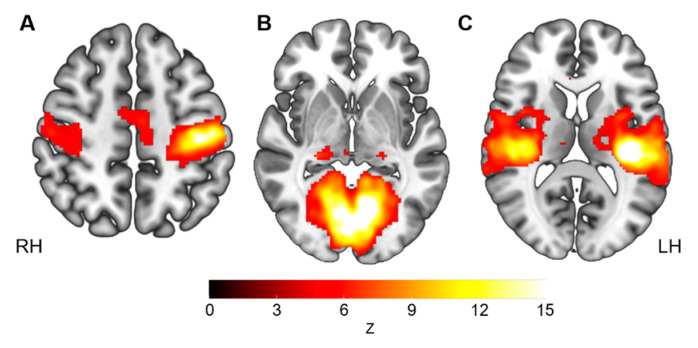
Group maps of the sensorimotor (**A**), visual (**B**) and auditory (**C**) networks for the youth with cerebral palsy, based on resting-state functional connectivity. Functional connectivity maps were constructed using a seed located in the (**A**) left precentral cortex (x = −41, y = −20, z = 62), (**B**) left lingual gyrus (x = −16, y = −74, z = −7) or (**C**) left Heschl gyrus (x = −40, y = −24, z = 9). Results are presented in radiological view.

After seeding the left precentral gyrus, we identified a typical sensorimotor network in the youth with CP. This network largely covered the pre- and post-central gyri, bilaterally, as well as the supplementary motor area (Figure 1A).

**Figure 2:**
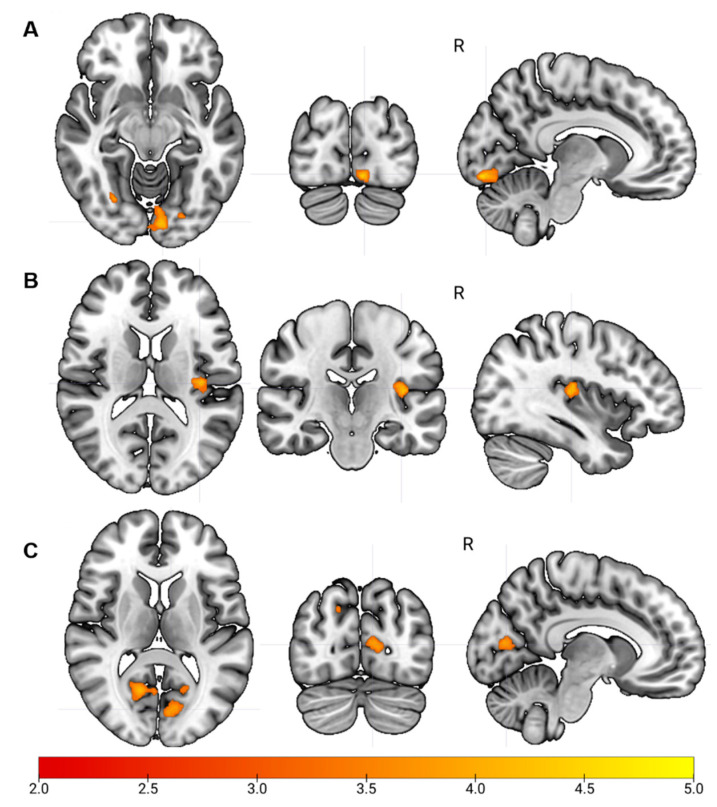
Clusters of significantly lower functional connectivity in the youth with CP relative to the controls. (**A**) Lower functional connectivity between the left precentral gyrus (seed) and the right cuneus; (**B**) lower functional connectivity between the left lingual gyrus (seed) and the right Heschl gyrus; (**C**) lower functional connectivity between the left Heschl gyrus (seed) and the right and left lingual gyri. Detail of the clusters can be found in Table 2. Significant threshold was set at a whole-brain level of *p* < 0.001 uncorrected, clusters > 20 voxels. The scale shows the z-score.

The two-sample *t*-test revealed four clusters with significantly lower FC in the youth with CP, compared with the controls (Table 2, Figure 2A). All the clusters were localized in the occipital lobe, covering the lingual and calcarine gyri, the cuneus and the superior occipital cortex. No significant clusters with higher FC in the youth with CP were detected.

#### 3.2.1. Visual Network Functional Connectivity

After seeding the left lingual gyrus, we identified a typical bilateral medial visual network in the youth with CP. This network largely and bilaterally covered the lingual gyri, calcarine and cuneus, as well as the middle and superior occipital gyri and the posterior section of the thalamus (Figure 1B).

The two-sample *t*-test revealed two clusters with significantly lower FC in the youth with CP, located in the left Heschl gyrus and the right rolandic operculum, compared to the controls (Table 2, Figure 2B). No significant clusters with higher FC in the youth with CP were detected.

#### 3.2.2. Auditory Network Functional Connectivity

After seeding the left Heschl gyrus, we identified a typical bilateral auditory network in the youth with CP. This network largely and bilaterally covered Heschl gyri, the Rolandic operculum, superior temporal gyri and the thalamus (Figure 1C).

The two-sample *t*-test revealed several clusters with significantly lower FC in the youth with CP compared to the neurotypical controls. Whereas the significant clusters were widespread across cerebrum and cerebellum (Table 2, Figure 2C), the majority of them were present in the occipital lobe, in the right and left calcarine and the left superior occipital gyrus. No significant clusters with higher FC in the youth with CP were detected.

### 3.3. Association with Spatiotemporal Gait Biomechanics

Consistent with the biomechanical literature, the youth with CP walked slower (CP = 0.929 ± 0.24 m/s; control= 1.16 ± 0.18 m/s; *p* = 0.0004), had a larger step width (CP = 0.147 ± 0.063 m; control = 0.089 ± 0.031 m; *p* = 0.00003) and had a shorter right (CP = 0.55 ± 0.09 m; control = 0.60 ± 0.08 m; *p* = 0.0001) and left (CP = 0.54 ± 0.11m; control = 0.67 ± 0.08 m; *p* = 0.00002) step length. There was no difference in the cadence between the respective groups (CP = 103.15 ± 24.6 steps/min; control = 103.43 ± 9.9 steps/min; *p* = 0.966).

**Figure 3:**
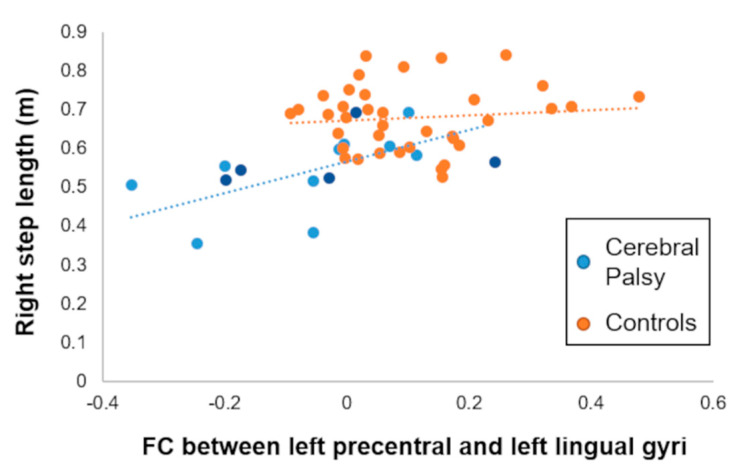
Association between resting-state functional connectivity (FC) and gait biomechanics. Correlation between the FC of the left precentral gyrus (seed) and right lingual cluster and the preferred average right step length. This association was significant in the CP group (*p*_FDR_ ≤ 0.05), but not in the neurotypical controls (*p* > 0.05). Light blue dots refer to the youth with spastic diplegic presentation, while the dark blue dots refer to the youth with a hemiplegic presentation. This association implies that youth with cerebral palsy who have weaker FC between the motor and occipital cortical areas tend to have a reduced step length.

Using the regions displaying a significant reduction of FC in the youth with CP, we sought to determine whether these observed FC differences were correlated with the noted aberrant gait biomechanics. For the group with CP, higher FC between the left precentral and lingual gyri was associated a larger right step length (ρ = 0.74, p_FDR_ = 0.04; Figure 3). This association remained significant after excluding the participants that had a hemiplegic presentation (ρ = 0.76, *p* = 0.01). We did not observe significant associations between the gait biomechanic variables and FC involving the other two seeds, even at an uncorrected threshold. Furthermore, none of these FCs were linked with the gait biomechanics seen in the controls (*p* > 0.05) or with the GMFCS level in the youth with CP (all *p* > 0.05).

## 4. Discussion

In this study, we found that youth with CP showed consistently lower functional connectivity among the three primary networks, particularly between the visual network and the sensorimotor and auditory regions. Furthermore, weaker functional connectivity between the precentral and the lingual gyri was associated with shorter step length in the youth with CP. Altogether, these results imply that altered connectivity between the motor and occipital regions might play a role in the uncharacteristic mobility seen in youth with CP. These results further fuel the impression that the brain functional abnormalities seen in youth with CP extent beyond the sensorimotor cortex. Further discussion of these findings is provided in the following paragraphs.

In neurotypical individuals, the SMN, AUD and VIS networks have been defined as part of the extrinsic system because they all respond to external stimuli [9,40] and show robust positive functional connectivity among the respective networks [11,13]. In contrast, our results demonstrated that youth with CP had mostly weaker connectivity among these three networks. First, this finding corroborates previous fMRI studies, which have shown that youth with CP have abnormalities that are not limited to the SMN but rather extend to other brain networks [26,28,41]. Second, this also suggests a potential reorganization of the brain extrinsic system, through a loss of functional integration. CP and other perinatal brain insults have been associated with neuronal plasticity not just in the lesioned area but also in intact brain areas [29,31,42]. Altered integration of both lesioned and non-lesioned primary motor cortices has also been reported in youth with CP using other neuroimaging modalities, such as task-based fMRI [43,44] and diffusion tensor imaging [30,45]. Together, these findings strongly underline the need of future studies on the neurophysiology of CP in the context of the whole-brain functional organization and the interdependency of the neural networks, especially those responding to external stimuli.

Another notable result of the study was the fact that the visual cortex was consistently found to be abnormally connected to the other two primary cortices (namely, the motor and auditory cortices). Both functional and structural studies have reported abnormalities related to the visual network [6,7,8,26,29] and visual tracks [30,45] in CP. Our finding supports the idea that the disconnectivity of the visual network from the other primary networks might reflect a pathophysiological mechanism leading to visual perception impairment in CP. In fact, visual dysfunction is now recognized as a core, co-occurring disorder affecting between 50% and 90% of individuals with CP [46,47,48,49]. This is also consistent with our previous works in MEG showing that children with CP had weaker cortical oscillations in the visual MT/V5 cortices and occipital cortices [6,7,8]. The altered connectivity seen between the motor and visual cortices might be partially a result of the white matter damage often seen in the peritrigonal region, as the optic radiation fibers are neighbors to the corticospinal tracts in this area [50]. Essentially, damage to these neighboring fiber pathways might impact the functional connectivity seen between the visual and motor cortices.

In contrast to previous rs-fMRI studies [26,27], we did not detect significantly higher FC emerging from the seed regions relative to the controls. Such differences are likely due to variances in the sample composition. Compared with the previous investigations, our study population was larger and slightly older than the sample in the previous investigations. During adolescence, it is well known that the brain networks undergo maturation and intense structural and functional reconfiguration [51,52,53]. For instance, in this age window, the SMN, which is a network that matures among the earliest, shows the reduction of functional connectivity with age [51]. In addition, it seems that the composition of the patient sample and the side and location of the seeds significantly impact the detection of higher FC in the patient groups [27]. A longitudinal examination of the functional integration of the SMN in CP on independent larger samples may provide a more definitive answer among these alternatives.

Lastly, we found positive associations between FC between the SMN and the VIS network and spatiotemporal gait biomechanics in the youth with CP. This implies that a greater FC between the motor-visual connections (i.e., higher SMN-VIS FC) is associated with a longer step length (i.e., closer to controls). This was only true for the right step length, which is likely related to the direct structural connections linking it to the left-sided precentral gyrus (hemisphere of the seed). Whereas it is fairly well accepted that the primary motor and somatosensory cortices areas are involved in the control of gait [54,55,56,57,58,59], our finding further supports the notion that impairment in the extrinsic system, not just in the SMN, may play a role in the extent of the motor dysfunction in youth with CP. This specific association may be related by the existence of physiologically relevant occipito-motor, but not auditory-motor, functional connections [60]. Several previous MEG studies have supported the idea that greater motor impairments may be directly linked with altered integration between sensorimotor and visuo-perceptual modalities in youth with CP [2,61,62]. Together with the current work, these results further support the premise that the uncharacteristic gait seen in youth with CP may be driven by a disconnectivity between the sensorimotor and visual networks [63]. Importantly, this finding also opens new doors, potentially identifying new functional biomarkers that could be used to measure the effectiveness of physical therapy and its impact on neuroplasticity of those with CP.

The current data should be considered in light of some limitations. First, our sample size was modest. However, the breadth of published studies in CP using rs-fMRI have also been composed of relatively small sample sizes, which reflects the difficulty in obtaining valid neuroimaging data in youth with CP. The smaller sample size also reflects our efforts to ensure that the significant associations were not driven by outliers, and we applied stringent multiple comparison correction (FDR) to prevent the identification of false positives. We also ensured that our main results remained significant when we excluded those participants with a hemiplegic presentation. Another possible limitation is that we used a seed-based approach, and results may vary with different seed locations [25,27] or another FC approach (i.e., independent component analysis) [28]. Since there was not a large hemispheric volume loss in our participants, we chose the seed location (i.e., left hemisphere) based on previously validated studies performed in large independent samples of healthy participants (for the motor and visual networks [39], for the auditory network [38]). We cannot exclude the possibility that some participants with CP may have had structural reorganization of their primary cortices that influenced the ideal location of the seed. However, we believe it is unlikely, because (1) we individually checked the location of each seed on the anatomical scan of each participant to ensure the correct location, and (2) we individually checked each individual and group FC map to ensure the identification of the networks of interest in the group with CP. Lastly, we acknowledge the relative heterogeneity of the sample with CP. Nevertheless, heterogeneity is typically the case for the population with CP.

## 5. Conclusions

This study suggests that youth with CP show a loss of functional integration between the three major brain networks responding to external sensorial stimuli. Furthermore, more abnormal gait biomechanics appear to be linked with a reduction in the functional connectivity between the sensorimotor and visual networks. Overall, the current findings support the emerging argument that there is significant overlap in the etiology of the motor and visual deficits in youth with CP. Lastly, these findings suggest rs-fMRI might be an important addition to other neuroimaging tools that can be used to better understand the plasticity of brain networks in relation to both the severity of CP and potentially rehabilitative outcomes.

## Figures and Tables

**Table 1:** Demographic information.

	Patients with Cerebral Palsy (n = 27)	Healthy Controls (n = 38)	Case-Control Differences (*p*-Value)
Age, mean (SD)	16.39 (4.90)	14.44 (2.35)	n.s.
Sex (%, N of females)	44.44 (12)	36.84 (14)	n.s.
Preferred Average Velocity, mean (SD, m/s)	0.93 (0.28)	1.17 (0.18)	<0.001
Preferred Average Cadence, mean (SD, steps/min)	100.64 (23.86)	103.73 (9.96)	n.s.
Preferred Average Left step length, mean (SD, m)	0.56 (0.12)	0.67 (0.08)	<0.001
Preferred Average Right step length, mean (SD, m)	0.55 (0.11)	0.68 (0.08)	<0.001
Preferred average width, mean (SD, m)	0.13 (0.06)	0.09 (0.03)	<0.001
Spastic diplegic CP (n, %)	17 (62.96)	-	
Hemiplegic CP (n, %)	10 (37.04)	-	

**Table 2:** Whole-brain functional connectivity differences between the neurotypical controls and youth with cerebral palsy.

Regions	Hemisphere	FC in CP	FC in Controls	Z	Cluster Size	x	y	z
		Mean (SD)	Mean (SD)					
**Seed: Left Precentral Gyrus (Sensorimotor Network)**								
**Contrast: Controls—CP**								
Lingual gyrus	R	−0.05 (0.16)	0.10 (0.13)	4.25	110	24	−72	0
Calcarine	L	−0.06 (0.17)	0.08 (0.11)	4.21	224	−8	−88	−12
Superior Occipital gyrus	R	−0.03 (0.20)	0.13 (0.17)	4.07	25	2	−40	−46
Cuneus	R	−0.05 (0.21)	0.07 (0.15)	3.87	37	2	−94	16
**Seed: Left Lingual Gyrus (Visual Network)**								
**Contrast: Controls—CP**								
Heschl gyrus	L	−0.02 (0.15)	0.17 (0.13)	4.00	105	−38	−22	12
Rolandic Operculum	R	−0.06 (0.16)	0.12 (0.15)	3.85	22	40	−14	24
**Seed: Left Heschl Gyrus (Auditory Network)**								
**Contrast: Controls—CP**								
Inferior parietal lobule	R	0.02 (0.15)	0.24 (0.15)	4.04	37	42	40	4
Cerebellum	L	0.03 (0.14)	0.25 (0.14)	3.90	30	−36	−36	−30
Calcarine	R	−0.02 (0.14)	0.12 (0.15)	3.88	141	16	−62	10
Frontal superior gyrus	R	−0.06 (0.09)	0.13 (0.17)	3.85	34	14	−4	78
Calcarine	L	0.01 (0.15)	0.19 (0.14)	3.84	315	−18	−62	8
Superior occipital gyrus	R	−0.08 (0.15)	0.06 (0.12)	3.49	24	18	−80	38
Superior occipital gyrus	L	−0.05 (0.18)	0.13 (0.14)	3.38	26	−22	−84	32

All coordinates are provided in MNI Space. FC are Z(r). CP= Cerebral Palsy.

## Data Availability

Data are available upon reasonable request to the corresponding author.
